# Impact of sociodemographic factors on COVID-19 survival: a nationwide 2,000,000 in-patients’ cohort in Brazil

**DOI:** 10.1590/1980-549720230050

**Published:** 2023-10-30

**Authors:** Raphael Mendonça Guimarães, Danielle Cristina Lourenço dos Santos Pastura

**Affiliations:** 1 Fundação Oswaldo Cruz, Escola Nacional de Saúde Pública Sergio Arouca – Rio de Janeiro (RJ), Brazil.

**Keywords:** Covid-19, Social determinants of health, Mortality, Survival analysis, COVID-19, Determinantes sociais da saúde, Mortalidade, Análise de sobrevivência

## Abstract

**Objective:**

To estimate the impact of sociodemographic factors on survival from COVID-19 in Brazil.

**Methods:**

Longitudinal data from a retrospective cohort of 2,000,000 hospitalizations due to COVID-19 in Brazil between March 2020 and May 2022, enrolled in SIVEP-Gripe, were analyzed.

**Results:**

The adjusted Cox model showed a 7% higher probability of death for men. 9% and 13% for the brown population compared to white and 16% for those living in the rural region. Long-lived elderly has a 301% higher probability when compared to young people.

**Conclusion:**

Sociodemographic factors interfere with survival from COVID-19 and should gain prominence in theoretical models and clinical aspects, and should be considered when formulating public policies, especially in countries with greater social inequality, such as Brazil.

## RESUMO

**Objetivo:**

Estimar o impacto dos fatores sociodemográficos para a sobrevida por COVID-19 no Brasil.

**Métodos:**

Foram analisados dados longitudinais de coorte retrospectiva de 2 milhões de internações por COVID-19 no Brasil entre março de 2020 e maio de 2022, arrolados no SIVEP Gripe.

**Resultados:**

O modelo ajustado de Cox mostrou probabilidade 7% maior de morte para os homens, 9 e 13% para a população parda em comparação à branca e 16% para aqueles que vivem na região rural. O idoso longevo tem probabilidade 301% maior quando comparado aos jovens.

**Conclusão:**

Os fatores sociodemográficos interferem na sobrevida pela COVID-19 e devem ganhar destaque nos modelos teóricos tais quais os aspectos clínicos, bem como ser considerados para formular políticas públicas, especialmente em países com maior desigualdade social, como o Brasil.

## INTRODUCTION

Current knowledge is consistent that structural factors that shape health inequities can be reflected in several outcomes associated with COVID-19, such as incidence, fatality, and mortality. Also, hospitalization data describe the clinical management of the most severe cases and express the opportunity for early diagnosis and treatment. Brazil was the epicenter of the COVID-19 pandemic in some phases over the last three years^1^.

Since the formalization of the pandemic status by the World Health Organization (WHO) until the moment when the Brazilian government announced the end of the State of Emergency in Public Health of National Importance, the country totaled over 700 thousand deaths, being the second country with the highest number of deaths^2^.

Current literature mainly contains cross-sectional studies that capture rapid diagnoses of health disparities related to COVID-19, even in countries that were the pandemic’s epicenter. Therefore, the aim of this study was to estimate the impact of sociodemographic factors on COVID-19 survival.

## METHODS

We concepted a retrospective cohort through secondary data from the Brazilian Database of Severe Acute Respiratory Syndrome (*Sistema de Informação da Vigilância Epidemiológica da Gripe* — SIVEP-Gripe). We included all COVID-19 database notifications from March 12^th^, 2020 (The date of the first death from COVID-19 in Brazil) until May 22^nd^, 2022 (when the government established COVID-19 was no longer an emergency of national interest). The database comprises 2,025,529 in-patients.

We evaluated as independent variables sex (male and female), age range (0 to 19 years old, 20 to 39 years old, 40 to 59 years old, 60 to 79 years old, and 80 years old and older), skin color (white, brown, and black), zone of residence (urban, peri-urban, and rural) and county of hospitalization (inhabitant or outsider). We obtained information on clinical characteristics that are potentially confounding to explain a worse prognosis or mortality, such as the occurrence of multimorbidity associated with higher mortality (simultaneous occurrence of 2 or more chronic conditions — heart disease, diabetes, obesity, asthma, and pneumopathies) and the occurrence of major symptoms (fever, dyspnea, low oxygen saturation (SpO_2_ < 95%), cough, respiratory distress, and fatigue). The study’s dependent variable was the duration of illness, between the occurrence of the first symptoms and the outcome (discharge due to cure or death from COVID-19). We excluded outliers, leaving 1,820,190 in-patients.

We performed a survival analysis. First, we used the Kaplan-Meier method to estimate the probability of survival at various time intervals. Based on these data, we applied the log-rank test to compare the survival curves considering cases of discharge due to cure and death. Since the objective was to describe the mortality attributed to COVID-19, we considered death from other causes as censoring. After applying the Kaplan-Meier method for the initial description, we performed a Cox regression to verify the magnitude of the associations. We calculated hazard ratios for each variable.

## RESULTS

In line with what the Centers for Disease Control and Prevention (CDC) consider risk factors for the severity of COVID-19, we explored the profile of hospitalized patients. Among those most at risk are people aged 60 years old and older (48.9%), severely obese people (BMI ≥40) (13.1%), immunocompromised people (6.0%), and people with other underlying medical conditions such as hypertension, diabetes, chronic kidney disease, and other chronic conditions (79.3%). Among people with other chronic conditions, 52.8% had more than 2 chronic diseases concomitantly.

The average time of disease progression was shorter among black men, with a gradient of association for older ages and residents of rural areas and those hospitalized in municipalities different from the cities of residence. Also, we identified that the probability of death was more remarkable for patients with multimorbidity who had major symptoms at admission (Table 1).

**Table 1:** Cox models of risk of death from Covid-19 in in-patients. Brazil, 2020–2022.

Characteristics	Categories	Descriptive			Cox Model			
		Kaplan-Meyer			Crude		Adjusted	
		Mean	SE	p-value (LogRank)	HR (95%CI)	p-value	HR (95%CI)	p-value
Sex	Female	17.01	0.01	<0.001	1	<0.001	1	<0.001
	Male	16.98	0.01		1.05 (1.04–1.06)		1.04 (1.03–1.05)	
Age (years)	0 to 19	24.63	0.09	<0.001	1	<0.001	1	<0.001
	20 to 39	21.12	0.04		1.23 (1.13–1.34)		1.22 (1.12–1.32)	
	40 to 59	18.99	0.02		1.51 (1.40–1.64)		1.47 (1.36–1.59)	
	60 to 79s	15.92	0.01		2.30 (2.12–2.49)		2.17 (2.00–2.35)	
	80+	12.85	0.02		3.75 (3.46–4.06)		3.59 (3.31–3.89)	
Skin color/race	White	17.07	0.01	<0.001	1	<0.001	1	<0.001
	Brown	16.42	0.01		1.09 (1.08–1.11)		1.10 (1.09–1.12)	
	Black	15.78	0.04		1.18 (1.16–1.21)		1.17 (1.15–1.20)	
Zone	Urban	17.04	0.01	<0.001	1	0.002	1	0.008
	Peri urban	16.27	0.17		1.14 (1.05–1.25)		1.14 (1.05–1.25)	
	Rural	15.87	0.04		1.16 (1.12–1.19)		1.15 (1.12–1.17)	
County of hospitalization	Inhabitant	17.01	0.01	0.005	1	0.006	1	0.018
	Outsider	16.99	0.02		1.08 (1.06–1.09)		1.01 (1.00–1.02)	
ICU care	No				1	<0.001	1	<0.001
	Yes				0.57 (0.56–0.58)		0.57 (0.56–0.58)	
Major symptoms	No	19.72	0.07	<0.001	1	<0.001	1	<0.001
	Yes	16.99	0,07		1.19 (1.16–1.23)		1.19 (1.15–1.23)	
Multimorbidity	No	17.20	0.03	<0.001	1	<0.001	1	<0.001
	Yes	15.73	0.02		1.16 (1.14–1.17)		1.15 (1.14–1.17)	

Source: SIVEP Gripe, Brazil.

Adjusted models showed a 7% higher probability of death for men. The longest-lived elderly has a 301% higher likelihood when compared to young people. Brown and black populations have a 9 and 13% higher probability, respectively, when compared to the white population. The rural population has a 16% higher risk of death, and people hospitalized outside their municipalities showed a marginal association of 1% with the risk of death. Risk estimates are statistically significant and consistent even when controlled for confounders, such as major symptoms at admission and multimorbidity. We also emphasize that the magnitude of the association of multimorbidity, a very relevant clinical characteristic, is also emphasized as a factor that serve as a proxy for socioeconomic status, such as area of residence and skin color/race. We performed the analysis using R (version 4.3.1).

## DISCUSSION

Almost three years after the outbreak of COVID-19 as a pandemic, the role of social inequalities is well established^3^. Historically, the most disadvantaged communities, especially in countries with greater social inequality, have had very different experiences during pandemics. Proper public policy responses depend on breaking structural inequalities. For this, the expansion of social protection is crucial. Public health needs to be associated with other policies aligned with social development^4^.

The more obscure prognosis of COVID-19 is directly related to the late detection of symptoms and associated comorbidities, especially those associated with microvascular and pulmonary complications^5^. However, the role of social determinants in the early detection of symptoms, as well as in the occurrence of chronic conditions such as heart disease, diabetes, and obesity, is remarkable. For this reason, sociodemographic factors such as age, sex, skin color/race, and place of residence have been studied worldwide. Regarding the relationship with the rural area, we highlighted that residents of rural areas face unique and complex challenges beyond those faced in urban areas. Rural locations have low testing rates and are therefore detecting disproportionately fewer cases of COVID-19 still in official stages. Additionally, inpatient services in rural areas or outlying areas are less able to meet demand^6^.

Using a quality database that enabled the construction of a cohort of more than 2 million people admitted to hospitals allowed an accurate diagnosis of this scenario. Its results align with the idea that COVID-19 is a syndemic, which reinforces the idea that there is a dynamic interaction between clinical aspects and social and environmental factors, ultimately resulting in a worse overall outcome^7^. The purpose of using the syndemic model is to examine the predictive ability of socioeconomic circumstances for the clinical course of the disease.

COVID-19 burdens are disproportionately high in underserved and vulnerable subpopulations. This situation typifies what Phelan et al^8^ call the fundamental causes of health. Simultaneously, structural inequities are responsible for increasing susceptibility to SARS-CoV-2 attack and increasing the likelihood of exposure to factors that exacerbate the disease, once it exists. Brazil has a deep structural inequality. In this sense, it is essential to discuss the implications of social determinants for health policies, not only so that there are advances in health promotion but so that we seek the availability of medical resources for the entire population^9^. In brief, the present study showed that racial and social determinants significantly influence COVID-19 survival, indicating that health policies must be coupled with public policies focused on reducing social and racial inequalities and improving access to health services by vulnerable populations.
